# Quantification of transmission risk in a male patient with a *FLNB* mosaic mutation causing Larsen syndrome: Implications for genetic counseling in postzygotic mosaicism cases

**DOI:** 10.1002/humu.23281

**Published:** 2017-07-06

**Authors:** Marie Bernkopf, David Hunt, Nils Koelling, Tim Morgan, Amanda L. Collins, Joanna Fairhurst, Stephen P. Robertson, Andrew G. L. Douglas, Anne Goriely

**Affiliations:** ^1^ Clinical Genetics Group MRC Weatherall Institute of Molecular Medicine University of Oxford Oxford United Kingdom; ^2^ Nuffield Department of Clinical Sciences Radcliffe Department of Medicine University of Oxford Oxford United Kingdom; ^3^ Wessex Clinical Genetics Service University Hospital Southampton NHS Foundation Trust Southampton United Kingdom; ^4^ Department of Women's and Children's Health Dunedin School of Medicine University of Otago Dunedin New Zealand; ^5^ Paediatric Radiology University Hospital Southampton NHS Foundation Trust Southampton United Kingdom; ^6^ Academic Unit of Human Development and Health, Faculty of Medicine University of Southampton Southampton United Kingdom

**Keywords:** FLNB, genetic counseling, Larsen syndrome, mosaicism, next‐generation sequencing, sperm

## Abstract

We report the case of a male patient with Larsen syndrome found to be mosaic for a novel point mutation in *FLNB* in whom it was possible to provide evidence‐based personalized counseling on transmission risk to future offspring. Using dideoxy sequencing, a low‐level *FLNB* c.698A>G, encoding p.(Tyr233Cys) mutation was detected in buccal mucosa and fibroblast DNA. Mutation quantification was performed by deep next‐generation sequencing (NGS) of DNA extracted from three somatic tissues (blood, fibroblasts, saliva) and a sperm sample. The mutation was detectable in all tissues tested, at levels ranging from 7% to 10% (mutation present in ∼20% of diploid somatic cells and 7% of haploid sperm), demonstrating the involvement of both somatic and gonadal lineages in this patient. This report illustrates the clinical utility of performing targeted NGS analysis on sperm from males with a mosaic condition in order to provide personalized transmission risk and offer evidence‐based counseling on reproductive safety.

Larsen syndrome (MIM# 150250) is an autosomal‐dominant congenital osteochondrodysplasia caused by mutations in the filamin B gene (*FLNB*) (Krakow et al., 2004). It is characterized by dislocations of large joints such as the hips, knees and elbows, talipes, scoliosis and cervical kyphosis, distinctive spatulate appearances of the distal phalanges, and characteristic craniofacial features such as a prominent forehead, depressed nasal bridge, malar flattening, and hypertelorism (Bicknell et al., 2007). Patients are frequently born with cleft palate, and conductive hearing loss arising from malformation of the ossicles is also common. As well as classic Larsen syndrome, individuals heterozygous for *FLNB* mutations can also have a number of lethal osteochondrodysplasia phenotypes such as atelosteogenesis type I (AOI; MIM# 108720) and type III (AOIII; MIM# 108721) and the especially severe boomerang dysplasia (MIM# 112310) (Bicknell et al., 2005; Farrington‐Rock et al., 2006). In addition, individuals homozygous or compound heterozygous for *FLNB* mutations encoding premature termination codons develop the recessively inherited condition spondylocarpotarsal syndrome (MIM# 272460), which is phenotypically distinct from the autosomal‐dominant disorders and is characterized principally by vertebral fusions (Krakow et al., 2004).

A few cases of apparent somatic and/or gonadal mosaicism have previously been reported in Larsen syndrome (Debeer, De Borre, De Smet, & Fryns, 2003; Frints, De Smet, Fabry, & Fryns, 2000; Petrella, Rabinowitz, Steinmann, & Hirschhorn, 1993). In such cases, as in other cases of disease due to somatic mosaicism, it is not usually possible to accurately determine the transmission risk to future offspring owing to a lack of quantitative information regarding the presence or absence of the mutation within the gamete pool. Here, we describe the case of a male patient with Larsen syndrome found to carry a relatively low‐level (∼10%) mosaic mutation in *FLNB* in multiple somatic tissues and in whom it was possible to provide personalized counseling on transmission risk by genetic testing using deep next‐generation sequencing (NGS) of a sperm sample.

A 23‐year‐old man presented to clinical genetics for further investigation of his childhood clinical diagnosis of Larsen syndrome. He had been born to healthy nonconsanguineous parents at 32 weeks’ gestation as a result of a dizygotic twin pregnancy—his unaffected twin brother being phenotypically nonidentical with different hair and eye color. There was no family history of skeletal dysplasia or any phenotype related to Larsen syndrome. He weighed 1 kg at birth, had a cleft palate (subsequently repaired) and required a tracheostomy until 3 years of age on account of having a “narrow airway.” He had bilateral conductive hearing loss due to ossicular fusion. Skeletally, his right side was more severely affected than his left, having had right congenital dysplasia of the hip, right knee subluxation, and requiring a right‐sided epiphysiodesis of the distal femur and proximal tibia. He also had bilateral talipes with calcaneovalgus deformities of both feet, which was noticeably more pronounced on the right. The toes of both feet had a shortened spatulate appearance, particularly marked on the right foot. Both hands were clinically normal to examination except for his right thumb, which had a markedly shortened distal phalanx with a spatulate appearance (Fig. 1). He had thoracic scoliosis and a prominent forehead and supraorbital ridge. He was of normal intelligence and his adult height was 1.72 m. On X‐ray, he was noted to have a wedge‐shaped vertebral body at T4. With respect to the pelvis, the iliac bodies were elongated and the ilia were constricted at the junction between the iliac wings and the iliac bodies. The femoral necks were relatively short and there was coxa vara on the right, with the height of the greater trochanter approaching the level of the acetabular roof. The sacrum was unremarkable with the exception of spina bifida occulta at S1, which is a common normal variant. There was shortening of the terminal phalanges of the fingers, present in both right and left hands but especially prominent in the right thumb, and in the right wrist an unusually shaped trapezium with fusion to the scaphoid and the absence of the hook of the hamate. There were no major malformations of the cervical spine, but the posterior elements exhibited minor anomalies and there was reversal of cervical lordosis leading to kyphosis.

**Figure 1:**
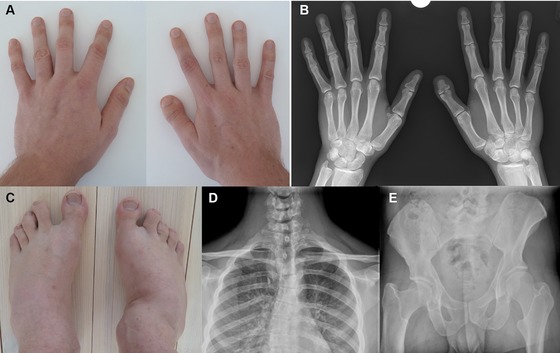
Clinical features. **A**: Spatulate appearance of the right thumb. **B**: X‐ray images of the hands showing mild shortening of both left and right distal phalanges, significantly shortened right first distal phalanx and right‐sided carpal abnormalities. **C**: Bilateral spatulate toes and calcaneovalgus foot deformity. Although both feet are involved, the deformity is markedly more pronounced on the right side. **D**: Wedge‐shaped T4 vertebral body, leading to thoracic scoliosis. **E**: Narrow iliac wings, shortened femoral necks, and right‐sided coxa vara

Given the right‐left asymmetric presentation of this patient who had been clinically diagnosed with Larsen syndrome, the presence of a postzygotic mosaic mutation was suspected. DNA extracted from peripheral blood had been tested 5 years earlier for mutations in exons 1–46 of *FLNB* using a high‐performance liquid chromatography WAVE system, but no mutation in *FLNB* had been identified using this method. As the patient remained keen to obtain a confirmatory molecular diagnosis for his condition and wished to clarify the potential transmission risk to future offspring, further tissue samples were collected. DNA extracted from cultured dermal fibroblasts and buccal mucosa (Supp. Method) was tested by dideoxy sequencing of exons 1–46 of *FLNB*, which allowed the identification of a c.698A>G variant in exon 4 (NM_001457.3) present at low level on the sequencing trace, consistent with the suspected mosaicism. This variant encodes a p.(Tyr233Cys) amino acid substitution that is predicted to be strongly deleterious (i.e., PolyPhen score = 1; SIFT score = 0) (Supp. Methods). This residue is located within the second calponin homology domain 2 (CHD2) of the filamin B protein, a known hotspot region for Larsen syndrome mutations (Fig. 2A).

**Figure 2:**
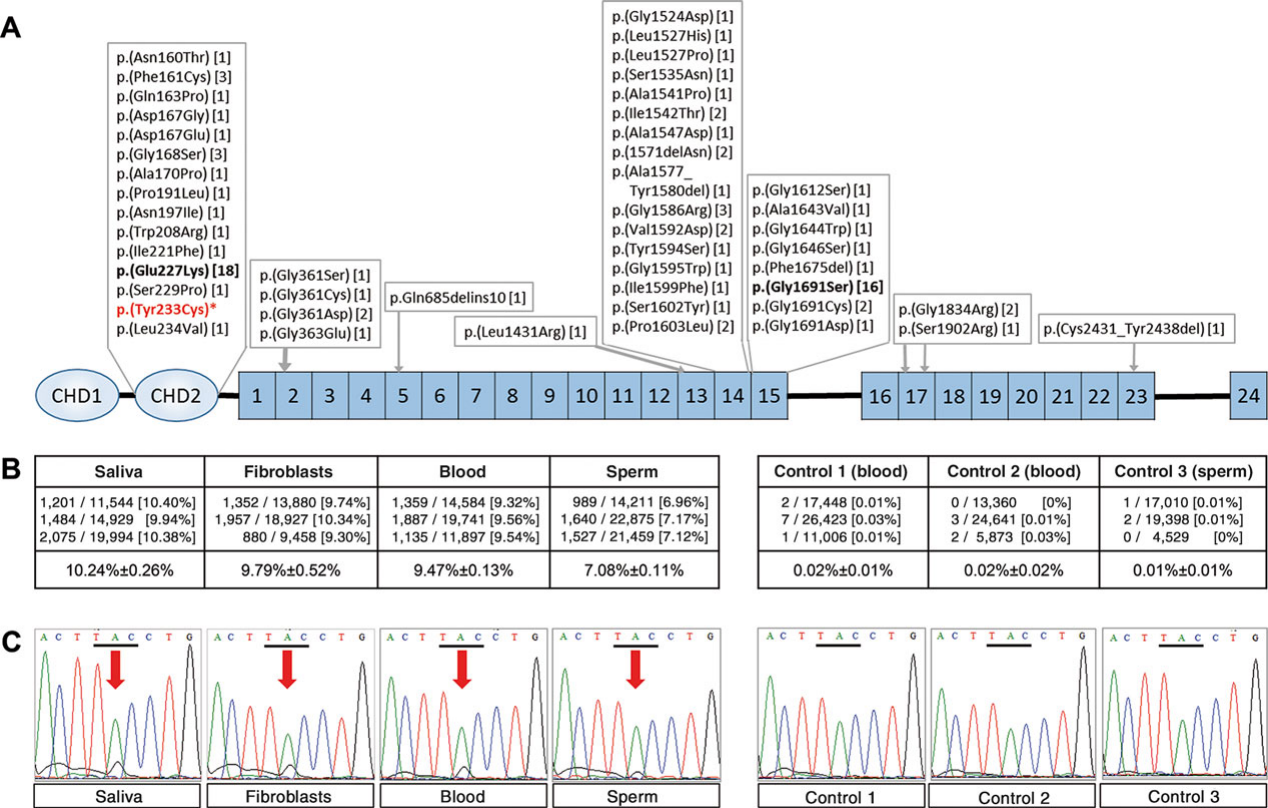
Quantification of the mosaic c.698A>G mutation in exon 4 of *FLNB*, encoding the p.(Tyr233Cys) substitution in multiple tissues. **A**: Schematic diagram of the filamin B (FLNB) (NP_001448.2) protein indicating the main protein domains, the location of the p.(Tyr233Cys) mutation (marked by an asterisk), and other previously reported mutations associated with Larsen syndrome (Bicknell et al., 2007; Daniel et al., 2012; Dobbs, Boehm, Grange, & Gurnett, 2008; Girisha et al., 2016; Krakow et al., 2004; Winer et al., 2009; Zhang et al., 2006). Numbers in brackets indicate the numbers of independently reported families with a given mutation; in bold are hotspot mutations. **B**: Table summarizing the counts of the c.698G allele and the total read counts obtained by deep NGS (with a quality score Q>30) at the chr3:58067414 mutation site for each of the triplicate PCR amplifications of the four patient samples (left) and three control samples (right). **C**: Dideoxy sequencing chromatograms confirming the presence of the c.698A>G substitution (arrow) at low levels in all four patient samples and its absence in the control samples. CHD, calponin homology domains located at the N‐terminal end of FLNB; boxes labeled “1–24” refer to structurally homologous filamin repeats

To obtain a precise quantification of the *FLNB* mutation levels and provide personalized counseling on transmission risk to offspring for this patient, DNA from four (blood, saliva, dermal fibroblasts, and sperm; the buccal mucosa DNA was not available) samples were amplified in triplicate and deep sequenced on the Illumina MiSeq platform (Supp. Methods). In parallel, three (blood or sperm) controls were also processed following the same procedure. A mean coverage depth of 16,000x (range: 4,529–22,875) was achieved at the c.698A>G mutation site. The mutation was detected in all four patient samples at levels ranging from 10.24%±0.26% in saliva, 9.79%±0.52% in fibroblasts, 9.47%±0.13% in blood, and 7.08%±0.11% in sperm (Fig. 2B). In all other locations of the 123‐bp amplified region, background mutation rates were below 0.15% for all samples (Supp. Fig.), whereas for control samples the mutation levels of position c.698A were estimated to be 0.02%±0.01% (Fig. 2B; Supp. Fig.). The presence of the c.698A>G mutation was further confirmed by dideoxy sequencing in all deep sequenced tissue samples from the patient (Fig. 2C).

In this report, we demonstrate the clinical utility of performing NGS testing of multiple somatic tissues and sperm in a male mosaic patient in order to accurately quantify mutation levels and establish his risk of transmitting the condition to future offspring. We originally identified a spontaneous mutation in *FLNB* (c.698A>G, encoding p.(Tyr233Cys)) that was detected at low level on dideoxy sequencing traces in DNA sampled from buccal cells and dermal fibroblasts of a Larsen syndrome patient. Although the p.(Tyr233Cys) mutation has not been reported previously, this substitution that is predicted to be deleterious affects a highly conserved amino acid in the CHD2, a region where other missense substitutions have been associated with Larsen syndrome (Fig. 2A) and is likely causative of the condition.

Dideoxy sequencing has traditionally been considered as the method of choice for diagnosis and validation of germline mutations. However, with a detection limit of ∼10%, it is now well recognized that this technology does not provide the sensitivity necessary for reliable detection of mosaic cases (Campbell, Shaw, Stankiewicz, & Lupski, 2015; Jamuar & Walsh, 2014). Using targeted NGS, deep sequencing can be achieved easily for a specific mutation and allows the determination of mosaicism levels with an unprecedented degree of accuracy. Using this approach, we detected the FLNB p.(Tyr233Cys) variant in all somatic tissues analyzed at levels ∼10%, confirming that in these tissues, ∼20% of cells are heterozygous for the mutation. Furthermore, we also analyzed a sperm sample from this patient and detected the mutation at ∼7% (i.e., 7% of the haploid sperm carried the mutation).

Observation of mixed mosaicism, involving both somatic and gonadal cells, indicates that the mutation originated at early stages of development, before the start of gastrulation, a time by which ∼10–15 mitotic divisions would have taken place in the embryo. Primordial germ cells are specified at around 2 weeks of development, signaling the separation of the germline‐soma lineages. Mutations that occur after this critical period will be confined to either the somatic or gonadal lineage. Soon after, the somatic lineage splits into three distinct germ layers (endoderm, mesoderm, and ectoderm) that will develop into specific tissues and organs (Campbell et al., 2015; De Felici, 2013; Rahbari et al., 2016). Hence, for cases where gonadal cells are not available (e.g., in females), aside from increasing the chance of sampling the relevant tissue for detection of a mosaic mutation, there may be some additional clinical benefits in quantifying mutation levels in somatic tissues of different embryonic origins. In this report, we sampled both mesoderm‐derived (dermal fibroblasts, blood, and saliva) as well as ectoderm‐derived tissues (buccal cells) and detected the variant in all samples, revealing the involvement of cells from different embryonic germ layers and pointing to an early postzygotic mutational event; this multitissue contribution may also be indicative of an increased risk of germline involvement, as was demonstrated in this report.

The use of NGS technology has, to the best of our knowledge, not been reported so far to provide personalized counseling on transmission risk and reproductive safety for patients with recognized postzygotic mosaicism. A few reports have documented the benefits of NGS analysis of sperm samples to quantify paternal gonadal mosaicism; however, these reports describe situations where mutation quantification was performed in clinically unaffected fathers following the birth of two siblings with the same condition (Shi et al., 2016) or in a case of suspected mixed somatic and gonadal mosaicism following the birth of an affected child (Tan et al., 2015).

The ability to quantify mutation levels in sperm and to provide personalized estimation of the transmission risk in postzygotic mosaic males prior to conception will likely impact upon clinical practice and a patient's own family planning decisions. In the absence of further information, genetic counseling of a patient with somatic mosaicism would advise that the transmission risk to offspring could be anywhere between 0% and 50%. Such an imprecise potential range of risk generates a high degree of uncertainty in patients. The specific risk of germline transmission in patients with a recognized somatic mutation depends on the timing of the original mutational event and whether it occurred before or after the separation of the germline‐soma lineages (occurring around 2 weeks of development). In cases of male somatic mosaicism, the use of NGS testing of sperm provides a direct, safe, and easy means to accurately quantify gonadal mosaicism levels and offers an evidence‐based estimate of transmission risk. This approach will allow couples to make informed decisions and, in cases where germline involvement can be ruled out, will reduce the need for potentially unnecessary interventions, such as in vitro fertilization and preimplantation diagnosis and/or prenatal testing, as well as alleviate the financial and emotional costs associated with such procedures.

## Supporting information

Supporting Material

Supporting Material
