# Supplementary material for: Quantification of transmission risk in a male patient with a FLNB mosaic mutation causing Larsen syndrome: Implications for genetic counseling in postzygotic mosaicism cases
Source: Hum Mutat. 2017 Jul 6;38(10):1360–4. doi: 10.1002/humu.23281 (PMC5638069; doi:10.1002/humu.23281)
Supplement: Supplementary file 1 — Supporting Material [file HUMU-38-1360-s001.pdf]

Supplementary Methods

DNA Samples

Patient samples from multiple tissues were collected, including buccal mucosa (obtained by a single swab of the left and right cheeks), cultured fibroblasts from skin (isolated from a punch skin biopsy of the right arm), saliva (collected using the Oragene system, DNA Genotek, ON, Canada), blood and sperm (full semen sample). DNA extraction of the semen sample was performed as previously described (Goriely, et al., 2003). Written informed consent was obtained for semen analysis, which was approved by the Oxfordshire Research Ethics Committee (OxREC C03.076). Other samples were collected with the patient's verbal consent on the basis of clinical diagnostic need.

Pathogenicity prediction of the c.698A>G (p.(Tyr233Cys) *FLNB* variant

The following pathogenicity prediction scores for the NM_001457.3(FLNB_i001):p.(Tyr233Cys) were obtained using ANNOVAR (version 2015-06-17 21:43:53) (Wang, et al., 2010):

SIFT_score = 0; Polyphen2_HDIV_score = 1; Polyphen2_HVAR_score = 1; LRT_score = 0; MutationTaster_score = 1; MutationAssessor_score = 3.93; LR_score = 0.967; VEST3_score = 0.964; CADD_raw = 4.741; CADD-Phred = 26.5; GERP++ RS = 5.73; phyloP46way_placental = 2.313; PhyloP100way_vertebrate = 9.236; SiPhy_29way_logOdds = 16.326.

This variant had not been described previously and is not present in publically accessible databases (ExAc, GnomAD, COSMIC70).

The FLNB p.(Tyr233Cys) variant has been deposited to the FLNB locus-specific database (LOVD 3.0 shared installation, DB-ID FLNB_00085) and can be accessed under URL: https://grenada.lumc.nl/LOVD2/mendelian_genes/variants.php?select_db=FLNB&action=view&view=0065321%2C0000200%2C0

Deep Next Generation Sequencing

Primers flanking the *FLNB* mutation site (chr3:58067414A>G (GRCh37/hg19)); c.698A>G (NM_001457.3) in exon 4 were designed and tailed with generic CS1 and CS2 sequence tags (indicated in lower cases): FLNB_CS1_Fw1: 5'-acactgacgacatggttctacaTGGGTGTTCATCCACCATGTCATT-3'; FLNB_CS2_Rev: 5'-tacggtagcagagacttggtctGCCCTGGCTTTCTTCGGGTTG-3'. Amplification of the *FLNB* mutation site was performed in triplicate for each of the four patient samples (blood, saliva, sperm, fibroblasts) and the three control samples in 20 µl PCR reaction using 30 ng genomic DNA and the High Fidelity Phusion Polymerase (New England Biolabs, MA, USA), following the manufacturer's recommendations for 28 cycles. To construct the Illumina library, a unique 10 bp barcode was added to each sample in a separate reaction, using: 1 µl of a 1:100 dilution of the previously amplified 212 bp PCR products, High Fidelity Phusion polymerase, 0.4 µM of each primer (PE1_CS1: 5'- AATGATACGGCGACCACCGAGATCTacactgacgacatggttctaca -3', PE2-BC-CS2: 5'- CAAGCAGAAGACGGCATACGAGATNNNNNNNNNNtacggtagcagagacttggtct -3'), where N_{10} represents the generic Illumina Barcode sequences for 10 cycles. The barcoded PCR amplicons were visualised on a 2% agarose gel and mixed in near-equimolar ratio. The final pool was purified using Agencourt AmPure XP Beads (Beckman Coulter, CA, USA) at a 0.8x (beads/PCR products) volume ratio following the manufacturer's instructions. The samples were sequenced on MiSeq platform (Illumina, CA, USA) with 2x151 paired-end reads using custom CS1-Seq primer (0.5 µM) for read 1 (5'- A+CA+CTG+ACGACATGGTTCTACA -3', where + indicates a LNA- (Locked Nucleic Acid) modified nucleotide, CS2-Seq primer for read 2 (5'- T+AC+GGT+AGCAGAGACTTGGTCT -3') and RC-CS2 primer for the Indexing read (5'-A+GAC+CA+AGTCTCTGCTACCGTA -3').

The raw sequencing reads were aligned to the GRCh37/hg19 reference genome using BWA-MEM Version 0.7.12-r1039 (Li and Durbin, 2009). The read counts for each nucleotide at each position of the sequenced amplicon with a minimum Q30 quality score (MAPQ/BASQ) were obtained using Samtools mpileup (Li, et al., 2009). The Supplementary Figure plots were generated in R using ggplot2 (Wickham, 2009). To further validate the presence of the mutation by dideoxy-sequencing in the patient samples, one of the triplicate PCR reactions for each tissue was sequenced with the FLNB_CS1_Fw2 primer.

References

Goriely A, McVean GA, Rojmyr M, Ingemarsson B, Wilkie AO. 2003. Evidence for selective advantage of pathogenic FGFR2 mutations in the male germ line. Science 301(5633):643-6.

Li H, Durbin R. 2009. Fast and accurate short read alignment with Burrows-Wheeler transform. Bioinformatics 25(14):1754-60.

Li H, Handsaker B, Wysoker A, Fennell T, Ruan J, Homer N, Marth G, Abecasis G, Durbin R, Genome Project Data Processing S. 2009. The Sequence Alignment/Map format and SAMtools. Bioinformatics 25(16):2078-9.

Wang K, Li M, Hakonarson H. 2010. ANNOVAR: functional annotation of genetic variants from high-throughput sequencing data. Nucleic Acids Res 38(16):e164.

Wickham H. 2009. Ggplot2 : elegant graphics for data analysis. New York: Springer.
