# Supplementary material for: Quantification of transmission risk in a male patient with a FLNB mosaic mutation causing Larsen syndrome: Implications for genetic counseling in postzygotic mosaicism cases
Source: Hum Mutat. 2017 Jul 6;38(10):1360–4. doi: 10.1002/humu.23281 (PMC5638069; doi:10.1002/humu.23281)
Supplement: Supplementary file 2 — Supporting Material [file HUMU-38-1360-s002.pdf]

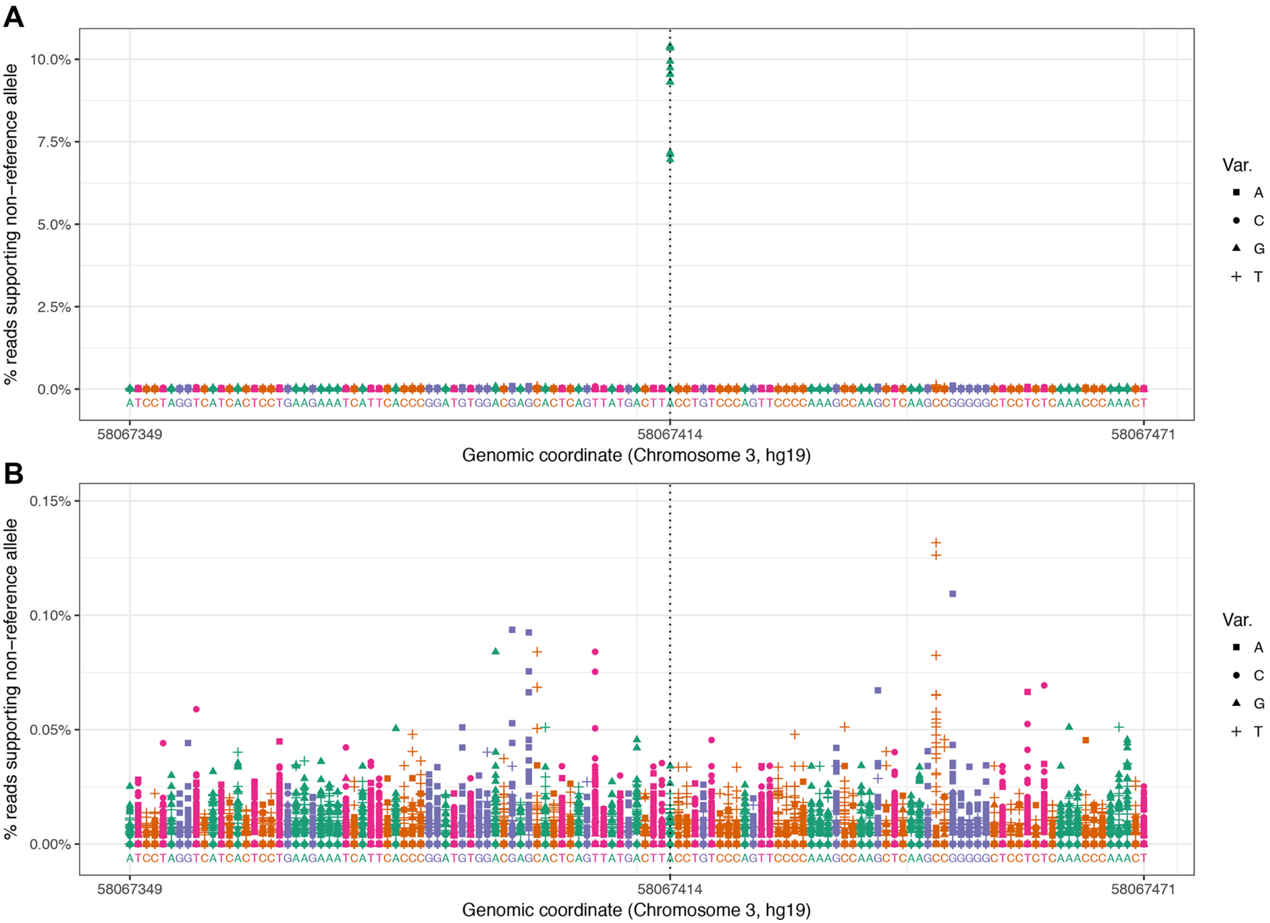


**Supplementary Figure**: Results of deep NGS sequencing of a 123bp amplicon (FLNB exon 4) flanking the mutation site (chr3:58067414A>G) in all analysed samples (including 3 replicates for each of the seven samples (4 patient samples and 3 control samples))

**(A)** Percentage of reads with non-reference base call for all samples across the full range of observed values.
**(B)** Higher magnification of the background levels (<0.15%) observed by deep NGS across the amplicon. Background mutation rates across the whole length of the amplicon are below 0.15% for all samples, while for control samples, the mutation levels for c.698G>A (dotted line) are below 0.03%.

Reference genome sequence and positions are indicated on the X-axis. Dotted vertical line represents the genomic position of the c.698G>A mutation in FLNB. Points are coloured by reference base, with their shape indicating the variant base found in the reads (as indicated on the key on the right of the plots)
